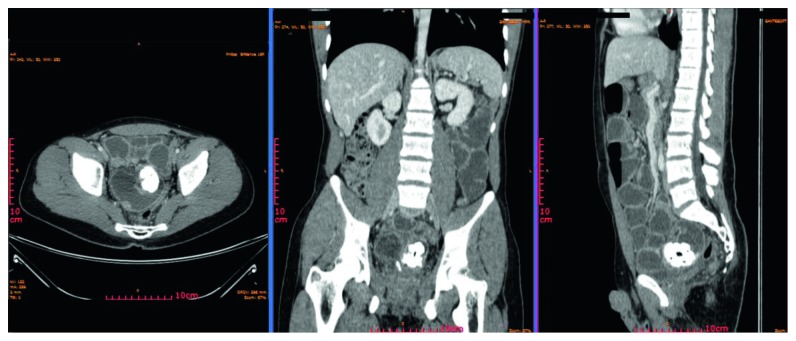# Large Enterolith Complicating a Meckel Diverticulum Causing Obstructive Ileus in an Adolescent Male Patient

**DOI:** 10.1155/2017/1871434

**Published:** 2017-12-17

**Authors:** Constantinos Nastos, Dimitrios Giannoulopoulos, Ioannis Georgopoulos, Christos Salakos, Dionysios Dellaportas, Ioannis Papaconstantinou, Theodosios Theodosopoulos, Georgios Polymeneas

**Affiliations:** ^1^Second Department of Surgery, National and Kapodistrian University of Athens, School of Medicine, Aretaieion University Hospital, Athens, Greece; ^2^First Department of Pediatric Surgery, National and Kapodistrian University of Athens, School of Medicine, Agia Sofia University Hospital, Athens, Greece; ^3^Department of Pediatric Surgery, National and Kapodistrian University of Athens, School of Medicine, Attikon University Hospital, Athens, Greece

## Abstract

We present a unique case of a 16-year-old male patient who was eventually diagnosed with a large enterolith arising from a Meckel's diverticulum. The enterolith had caused intermittent intestinal symptoms for three years before resulting in small bowel obstruction requiring surgical intervention. Meckel's enterolith ileus is very rare with only few cases described in the literature. To our knowledge, this is only the second case of Meckel's enterolith which had caused intermittent symptoms over a period of time, before resulting in ileus, and the first case where the intermittent symptoms lasted several years before bowel obstruction. The patient had been evaluated with colonoscopy, computerized tomography (CT), and magnetic resonance imaging enterography (MRIE); a calcified pelvic mass had been found, but no further diagnosis other than calcification was established. The patient presented at our emergency department, with symptoms of obstructive ileus and underwent exploratory laparotomy, where a large enterolith arising from a Meckel's diverticulum (MD) was identified, causing the obstruction. A successful partial enterectomy, enterolith removal, and primary end-to-end anastomosis took place; the patient was permanently relieved from his long-standing symptoms. Consequently, complications of Meckel's diverticulum and enterolithiasis have to be included in the differential diagnosis of abdominal complaints.

## 1. Introduction

Meckel's diverticulum is a common embryological remnant that can be found incidentally during surgery for other pathology, or may manifest as acute abdomen, most commonly due to diverticular inflammation. More rare complications of Meckel's diverticulum, including bleeding, herniation, intussusception, and enterolithiasis, have also been reported [1, 2]. We present a rare case of a large enterolith formed inside a Meckel's diverticulum that was diagnosed in adolescence after having caused intermittent abdominal symptoms and finally small bowel obstruction.

## 2. Case Presentation

**Figure 1:**
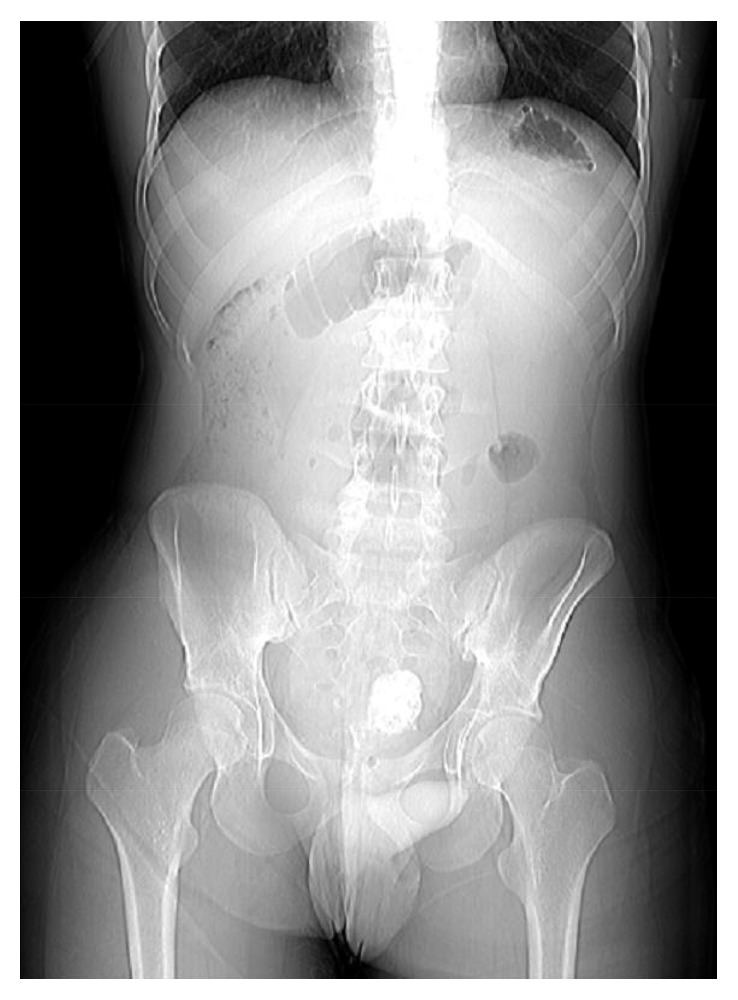


A 16-year-old patient presented to our outpatient clinic for evaluation of a pelvic calcified mass, initially found three years earlier in an abdominal X-ray, during investigation of an episode of lower right quadrant pain (Figure 1).

At that time, the patient did not require any surgical intervention and was managed conservatively. He was advised to have an outpatient workup of this mass. He was evaluated by a gastroenterologist and underwent an MRI enterography and colonoscopy. The MRI enterography revealed a calcified lesion in the distal ileum, located inside the pelvis, while the colonoscopy failed to recognize any lesion or inflammation in the terminal ileum. Meanwhile, the patient occasionally complained of nonspecific abdominal symptoms, which did not prompt him to seek further medical attention and resolved automatically.

Two years later, we scheduled an abdomen computerized tomography (CT), as a follow up. Contrast-enhanced abdominal CT revealed the same calcified lesion, 5 cm in diameter, that was in contact with the small bowel in the pelvis. The lesion had not grown in size during the last years.

Three years after the first onset of symptoms, the patient presented in our emergency department with symptoms of small bowel obstruction, namely, vomiting, intense colicky abdominal pain, and gas/flatus retention for 48 hours. Clinical examination revealed abdominal tenderness, distension, and hyperactive bowel sounds upon auscultation. Lab studies revealed mild leukocytosis. Plain X-ray showed multiple air-fluid levels. A new CT scan of the abdomen revealed obstruction of the small intestine, by the aforementioned mass (Figure 2).

The patient underwent an emergency laparotomy, where a loop of small bowel was found to be adherent to the pelvic wall. Upon dissection and mobilization of this loop from the pelvic wall, an enlarged and inflammed Meckel's diverticulum was identified. The diverticulum contained an enterolith, which was obstructing the bowel lumen. A partial enterectomy and an end-to-end anastomosis of the small bowel were performed.

The operation led to resolution of the obstruction, and the postoperative course was uneventful.

The pathology report confirmed the presence of a large enterolith, 5 cm in diameter, and ulcerative transmural inflammation of the resected part of the small intestine.

Six months following the operation, the patient was completely relieved from his occasional abdominal complaints and remained totally asymptomatic.

Informed consent was obtained from the patient for publication of this case report.

## 3. Discussion

Meckel's diverticulum is usually asymptomatic (85–95%), but clinical presentations associated with Meckel's diverticulum are diverticulitis, rectal bleeding, and small intestinal obstruction [3]. Small bowel obstruction can be the result of intussusception, volvulus around a fibrous band between Meckel's diverticulum and umbilicus, internal hernia through a fibrous band or an aberrant vitelline artery, prolapse of Meckel's diverticulum through a persistent omphalomesenteric effect, Littre's hernia, and rarely, enterolithiasis [1, 2, 4].

Meckel's diverticulum is complicated with enterolithiasis in 3–10% of cases [1]. In a large study by Park et al. involving 1476 patients with Meckel's diverticulum, it was found that 0.7% of asymptomatic patients and 6% of symptomatic patients had enterolithiasis at laparotomy [5]. The differential diagnosis of enterolithiasis includes calcified abscess, possibly due to Crohn's disease, possibly ingested foreign body, phytobezoar, trichobezoar, lactobezoar or pharmacobezoar, calcified neoplasm, undescendant testicle, teratoma, and abscess due to Crohn's disease [1].

Enteroliths can cause obstruction [1], diverticulitis [6], injury to the bowel mucosa [1], perforation [1], afferent loop syndrome [7], intussusception [8], gangrene [1], hemorrhage [1], and iron deficiency anemia. Meckel's diverticulum-related enterolith intestinal obstruction has rarely been reported [4].

To our knowledge, our case is only the second to describe a Meckel associated large enterolith that had been causing intermittent-type bowel symptoms for a certain period of time, before resulting in acute abdomen, due to obstructive ileus, thus requiring immediate surgical intervention [9]. Interestingly, this is the first case that the intermittent symptoms had lasted for several years, without a definite diagnosis regarding the cause of the symptoms being made in the meanwhile, before eventually producing small bowel obstruction.

In conclusion, enterolithiasis is an entity that can be a diagnostic challenge for the clinician. Meckel diverticulum-associated enterolithiasis can produce bowel obstruction. The clinician should include Meckel's diverticulum complications and enterolithiasis in the differential diagnosis of abdominal symptoms, either acute or chronic.

## 4. Conclusions

Meckel diverticulum-associated enterolithiasis is a rare condition that can be either asymptomatic or produce nonspecific symptoms but can also manifest itself as acute abdomen. Intestinal obstruction due to Meckel's enterolithiasis is very rare, with only few cases described in the literature. Our patient experienced intermittent symptoms for a course of several years before developing intestinal obstruction. Our case underlines that complications of Meckel's diverticulum and Meckel diverticulum-associated enterolithiasis need to be included in the differential diagnosis of abdominal complaints, as this diagnosis can be particularly challenging.

## Figures and Tables

**Figure 2:**